# Corneal perforation associated with ocular graft-versus-host disease

**DOI:** 10.3389/fonc.2022.962250

**Published:** 2022-09-15

**Authors:** Yue Xu, Ying-Ming Wang, Zheng-Tai Sun, Xiao-Long Yang, Xin-Yu Zhuang, Ya-Ru Ren, Ying-Jie Chen, Feng Chen, Xiao Ma, Xiao-Wen Tang, Xiao-Feng Zhang

**Affiliations:** ^1^ Department of Ophthalmology, Dushu Lake Hospital Affiliated to Soochow University, Suzhou, China; ^2^ Department of Ophthalmology, First Affiliated Hospital of Soochow University, Suzhou, China; ^3^ National Clinical Research Center for Hematologic Diseases, Jiangsu Institute of Hematology, The First Affiliated Hospital of Soochow University, Suzhou, China; ^4^ Institute of Blood and Marrow Transplantation, Collaborative Innovation Center of Hematology, Soochow University, Suzhou, China

**Keywords:** corneal perforation, ocular graft-versus host disease, corneal ulceration, keratoplasty, conjunctival flap covering surgery

## Abstract

Corneal perforation is a rare and serious complication of ocular graft-versus-host disease (oGVHD) patients. This study was to retrospectively report seven corneal perforation patients after allogeneic hematopoietic stem cell transplantation (HSCT). Demographic, hematologic, and ophthalmological data of patients were clarified in detail. Nine eyes of seven corneal perforation patients were clarified (Cases 3 and 6 were bilateral and the others are unilateral). All the cases had other affected GVHD organs, especially skin involvement. The duration between HSCT and corneal perforation was usually long with 21 (17–145) months as median interval, whereas the duration between oGVHD diagnosis and corneal perforation was relatively shorter with 4 (2–81) months as median interval. Most patients presented to ophthalmology department with poor visual acuity, BUT and Schirmer’s test. Eyelid marginal hyperemia and irregularity were observed in most corneal perforation eyes. Keratoplasty or conjunctival flap covering (CFC) surgeries was performed after corneal perforation. After a long-term follow-up for most patients (median 21 months, range: 2–86 months), only two eyes of two patients (22.22%) had a final BCVA of 20/100 or better. Patients involved in both cutaneous GVHD and blepharitis indicate the aggressive development of oGVHD. Early diagnosis, long-term follow-up, and effective multi-disciplinary treatments for oGVHD patients are essential. Corticosteroids and immunosuppressor remain essential, whereas the use of topical corticosteroids should be carefully considered in corneal ulceration patients. In addition, appropriate surgeries should be performed to control oGVHD development in time.

## Introduction

Allogeneic hematopoietic stem cell transplantation (HSCT) has become the standard method for various hematopoietic malignancies, autoimmune, and hereditary disorders (1). Over 50,000 patients undergo HSCT every year worldwide (2). Graft-versus-host disease (GVHD) is the major complication of HSCT and limits the chances of HSCT success, which predominantly involves in lungs, skin, mouth, gastrointestinal tract, and eyes (3). Ocular GVHD (oGVHD), as a frequent manifestation in chronic GVHD, occurs in 30–60% of HSCT patients and may be the indicator for poor mortality and major occurs in cornea (1, 4). Dry eye disease or keratoconjunctivitis sicca represents the hallmark of oGVHD and may result in corneal neovascularization, corneal ulceration and perforation (5).

Similar to dry eye disease, oGVHD is considered to be characterized by chronic inflammation and pathogenic fibrosis for now (6). As a result, the treatment strategies of oGVHD are focus on ocular surface lubrication, tear preservation, and inflammation reduction, such as topical corticosteroids, immunosuppression, and autologous serum eye drops and contact lenses (7). For severe patients with corneal ulceration and perforation, keratoplasty or conjunctival flap covering (CFC) surgery may be needed to prevent aggressive vision loss (4).

Although the high incidence of oGVHD in HSCT patients, the outcomes are diverse, which may influence vision-related quality of life and result in sight-threatening complications in extreme cases (8). Hence, this study focuses on oGVHD patients with corneal perforation to give a better insight into the clinical courses and provide effective treatment strategies for these patients.

## Materials and methods

### Demographic and hematologic data of patients

A total of 252 HSCT patients who presented to ophthalmology department of Dushu Lake Hospital Affiliated to Soochow University and First Affiliated Hospital of Soochow University from 1 August 2012 to 31 December 2021 were retrospectively enrolled and analyzed. All participants were Han ethnicity. Patient gender, age at first presentation to ophthalmology department, hematologic disease for HSCT, age at time of HSCT, donor data, other affected organs related to GVHD at first presentation to ophthalmology department were recorded in detail.

### Ophthalmological data and oGVHD diagnosis

Ophthalmological data including visual acuity, intraocular pressure, corneal fluorescein staining scores, breakup time of tear film (BUT), and Schirmer’s test without anesthesia were also recorded for 252 HSCT patients. According to National Institutes of Health Consensus Conference 2014 criteria, the diagnosis of oGVHD patients was depending on Schirmer’s test and slit-lamp examination (9). The HSCT patients with Schirmer test ≤5 mm/5 min or Schirmer test 6–10 mm/5 min as a result of other causes and keratoconjunctivitis sicca by slit-lamp examination were considered as oGVHD patients.

Among these oGVHD patients, the patients who developed corneal perforation associated with oGVHD were selected out. The best-corrected visual acuity (BCVA), fluorescein staining scores, BUT, and Schirmer’s test at first presentation to ophthalmology, BCVA, marginal hyperemia, and marginal irregularity at corneal perforation, BCVA at last follow-up, surgery details, use of topical immunosuppression, and corticosteroid regimens in the 2 months prior to corneal perforation in the affected eyes of these oGVHD patients were further collected and analyzed in this study. Time courses of HSCT, oGVHD diagnosis, corneal perforation, surgeries, and last follow-up condition in these affected eyes were calculated based on their medical records.

For patients who accepted keratoplasty, temporary blepharorrhaphy was also performed during the surgery and the stitches were taken out after 1 week. All these affected eyes utilized 50% autologous serum eye drops in the first 3 months after keratoplasty and reduced to 30% autologous serum eye drops after 3 months. Tacrolimus, fluorometholone, and ofloxacin eye ointment were also used in these eyes after keratoplasty. The sizes of keratoplasty and histopathological results were reviewed. Histologic sections of the corneas were processed for staining with hematoxylin and eosin.

## Results

### Demographic data of oGVHD patients

A total of 198 patients (including 122 men and 76 women) out of 252 HSCT patients (including 151 men and 101 women) were diagnosed as oGVHD patients, whereas the other 54 patients did not meet the diagnostic criteria at first presentation to ophthalmology department. The median age of 198 oGVHD patients was 33 (range: 4–58) years old.

Within these 198 oGVHD patients, nine eyes of seven patients (3.5%, Cases 3 and 6 were bilateral corneal perforation and the other five patients are unilateral cases) developed corneal perforation during the follow-up of oGVHD. All the seven patients had not undergone ocular surgeries or suffered other ocular diseases (such as dry eye syndrome, glaucoma, and retinal diseases) except refractive error prior to HSCT. The demographic and hematologic data of these seven patients were summarized in **Table 1**. The median age of seven oGVHD patients was 30 (range: 16–39) years old, and most of these patients were men (except Case 3, 85.71%). The hematological malignancies were varied, including three acute lymphoblastic leukemia (ALL) patients (42.86%), two non-Hodgkin lymphoma (NHL) patients (28.57%), one acute myeloid leukemia (AML) patient (14.29%), and one chronic myeloid leukemia (CML) patient (14.29%). In addition, this cohort included four male recipients of male donors (57.14%), two male recipients of female donors (28.57%), and one female recipient of male donor (14.29%). The HLA matching between donors and recipients of this cohort contains HLA identical (four patients, 57.14%) and HLA haploidentical (three patients, 42.86%). Notably, all the seven cases had other affected organs related to GVHD, especially skin involvement.

**Table 1:** Demographic and hematologic data of corneal perforation patients.

Case	Gender	Age at time of HSCT (years)	Diagnosis	Donor			Other affected organs related to GVHD
				Gender	Relationship	HLA matching	
1	Male	19	ALL	female	mother	HLA haploidentical	gastrointestinal tract, skin
2	Male	27	NHL	male	brother	HLA identical	gastrointestinal tract, skin, mouth, liver, gallbladder, lungs
3	Female	30	ALL	male	brother	HLA haploidentical	skin, mouth, lungs
4	Male	16	ALL	male	father	HLA haploidentical	skin
5	Male	39	NHL	female	sister	HLA identical	skin
6	Male	31	AML	male	brother	HLA identical	skin, mouth
7	Male	35	CML	male	brother	HLA identical	mouth, liver

hematopoietic stem cell transplant (HSCT), graft-versus-host disease (GVHD), acute lymphoblastic leukemia (ALL), non-Hodgkin lymphoma (NHL), acute myeloid leukemia (AML), chronic myeloid leukemia (CML), human leukocyte antigen (HLA).

### Ophthalmological data of oGVHD eyes and histopathology results

BCVA at different time points of oGVHD clinical courses, fluorescein staining score, BUT, and Schirmer’s test at first presentation to ophthalmology, marginal hyperemia, and irregularity status at corneal perforation, ophthalmologic surgeries performed, topical immunosuppression, and corticosteroid regimens in the 2 months prior to corneal perforation in the affected eyes were shown in **Table 2**. In the seven eyes of five patients with initial BCVA records (except Cases 2 and 7), most patients presented to ophthalmology department with poor visual acuity. BCVA was 20/32 at initial presentation in the right eye of Case 4, whereas BCVA was 20/40 or worse at initial presentation in the other six eyes of four patients (Cases 1, 3, 5, and 6). The fluorescein staining scores were more than 3 in most patients. Both the BUT and Schirmer’s test were terrible in most patients, which were similar to other severe oGVHD patients. At the time of corneal perforation, BCVA was consistently poor, with the best BCVA being 20/100 and three eyes of three patients having count fingers or worse. Notably, worse eyelid marginal hyperemia and irregularity were observed in most corneal perforation eyes. After a long-term follow-up for most patients (median: 21 months, range: 2–86 months), only two eyes of two patients (22.22%) had a final BCVA of 20/100 or better. Eight eyes of six patients (except Case 2) underwent keratoplasty surgeries with transplantation bed sizes ranging from 2.00 to 8.00 mm. Only Case 4 also used amniotic membrane before corneal perforation to control the corneal dissolution development. Four patients (Cases 3, 4, 5, and 7, 57.14%) utilized both topical immunosuppression eye drops (tacrolimus or cyclosporine) and topical corticosteroid eye drops (dexamethasone or fluorometholone) in the 2 months prior to corneal perforation. Case 2 did not follow standard treatment in ophthalmology department and only used artificial tears eye drops sometimes in the 2 months prior to corneal perforation. Cases 1 and 6 utilized topical immunosuppression and corticosteroid eye drops, respectively, in the 2 months prior to corneal perforation.

**Table 2:** Ophthalmological data, surgeries, topical immunosuppression, and corticosteroid regimens in the 2 months prior to corneal perforation in affected eyes.

Case	Eye	At first presentation to ophthalmology				At corneal perforation			At last follow-up visit	Type of surgery	Size of keratoplasty	Topical immunosuppression	Topical corticosteroid
		BCVA	Fluorescein staining score	BUT	Schirmer’s test	BCVA	Marginal hyperemia	Marginal irregularity	BCVA				
1	OD	20/63	3	0	1	20/160	3	1	20/63	keratoplasty	2.00 mm	Tacrolimus	/
2	OD	UNK	UNK	UNK	UNK	20/125	3	2	20/160	CFC	/	/	/
3	OD	20/40	4	0	2	20/400	3	2	CF/30cm	keratoplasty+CFC	4.00 mm	Tacrolimus	Dexamethasone
	OS	20/40	5	0	1	CF/30 cm	3	2	20/400	keratoplasty+CFC	3.00 mm	Tacrolimus	Dexamethasone
4	OD	20/32	3	0	2	20/100	2	1	20/40	CFC+keratoplasty	4.00mm	Tacrolimus	Fluorometholone
5	OD	20/40	4	0	0	HM	3	2	HM	keratoplasty	6.00 mm	Cyclosporine	Fluorometholone
6	OD	20/100	5	0	3	20/100	3	0	CF/30cm	keratoplasty	3.50 mm	/	Dexamethasone
	OS	20/250	5	0	2	20/250	3	0	20/500	keratoplasty	4.00 mm	/	Dexamethasone
7	OS	UNK	UNK	UNK	UNK	HM	2	1	HM	keratoplasty	8.00 mm	Tacrolimus	Fluorometholone

breakup time of tear film (BUT), best-corrected visual acuity (BCVA), the right eye (OD), the left eye (OS), unknown (UNK), count fingers at 30 cm (CF/30cm), hand motion (HM), conjunctival flap covering surgery (CFC).

**Figure 1:**
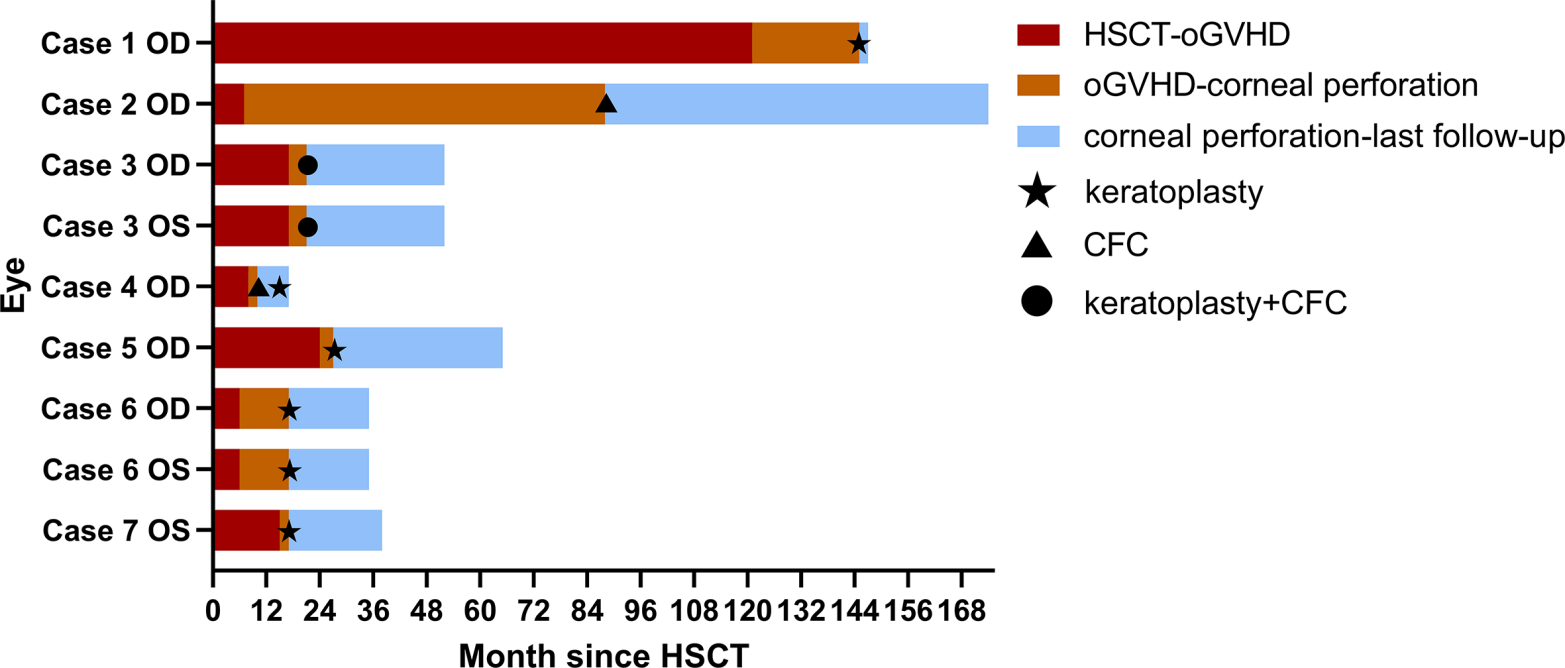
Time courses of HSCT, oGVHD diagnosis, corneal perforation, surgeries, and last follow-up in each affected eye of seven patients. The duration between HSCT and oGVHD diagnosis was shown in red. The duration between oGVHD diagnosis and corneal perforation was presented in orange. The duration between corneal perforation and last follow-up in ophthalmology department was shown in blue. Five affected eyes in Cases 1, 5, 6, and 7 underwent keratoplasty surgeries at the time of corneal perforation. The right eye of Case 2 underwent CFC at the time of corneal perforation. Both eyes of Case 3 were found corneal perforation at the same time and accepted keratoplasty and CFC combined surgery. The right eye of Case 4 accepted CFC at the time of corneal perforation and underwent keratoplasty 5 months later because of oGVHD progression during the follow-up. Abbreviations: the right eye (OD), the left eye (OS), hematopoietic stem cell transplant (HSCT), ocular graft-versus-host disease (oGVHD), conjunctival flap covering surgery (CFC).

Time courses of HSCT, oGVHD diagnosis, corneal perforation, surgeries, and last follow-up in each affected eye of seven patients were illustrated in **Figure 1**. Generally, the affected eyes had short-time intervals between oGVHD diagnosis and corneal perforation, with the median interval of 4 (range: 2–81) months. The median interval between HSCT and oGVHD diagnosis was 15 (range: 6–121) months, and the median interval between HSCT and corneal perforation was 21 (range: 17–145) months.

Five affected eyes in Cases 1, 5, 6, and 7 underwent keratoplasty surgeries at the time of corneal perforation. The right eye of Case 2 underwent CFC at the time of corneal perforation. Both eyes of Case 3 were found corneal perforation at the same time and accepted keratoplasty and CFC combined surgery. The right eye of Case 4 accepted CFC at the time of corneal perforation and underwent keratoplasty 5 months later because of the retraction of conjunctival flap and progressive corneal dissolution during the follow-up.

Histopathology of corneas in Cases 4, 5, and 7 after keratoplasty was presented in **Supplementary Figure 1**. A small amount of squamous epithelium and collagen fibrous stroma were seen in Case 4. Hemorrhage and inflammatory cell infiltration were seen in Case 5. Collagenization of a small amount of fibrous tissue was seen in Case 7.

**Figure 2:**
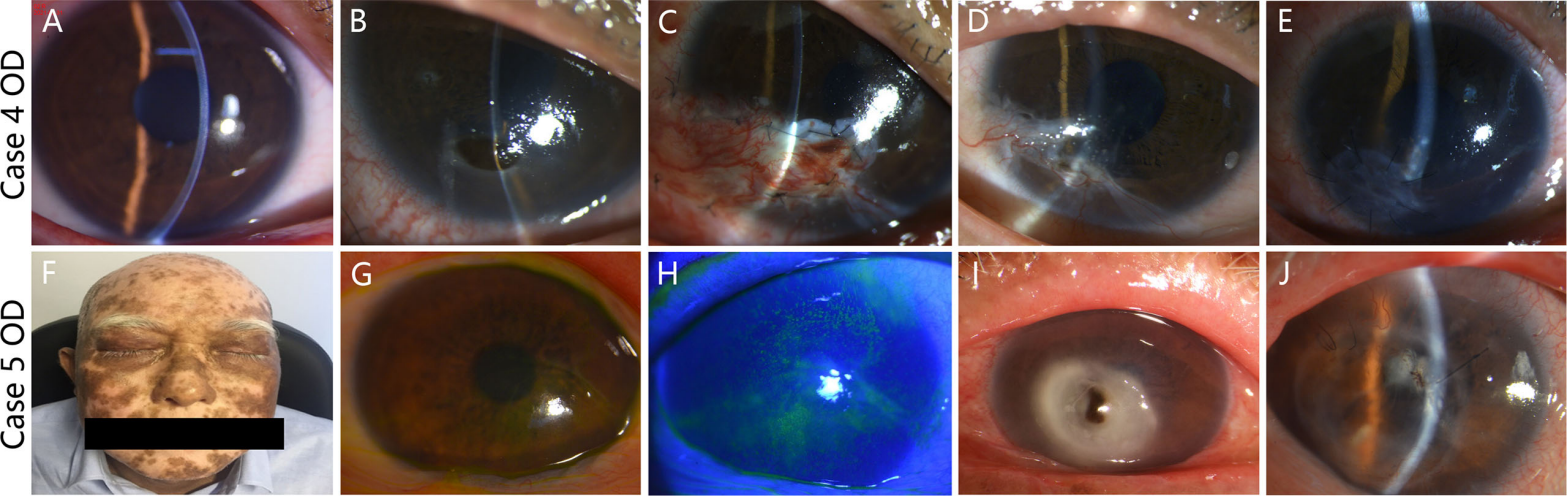
oGVHD development in Cases 4 and 5. Slit lamp photographs at the time of first presentation to ophthalmology department **(A)**, corneal perforation **(B)**, post-CFC surgery **(C)**, conjunctival flap retraction and corneal dissolution progression **(D)**, and post-keratoplasty surgery **(E)** in Case 4 were presented. The BCVAs of Case 4 at first presentation, corneal perforation, and last follow-up visit were 20/32, 20/100, and 20/40, respectively. Meanwhile, Case 5 had vitiligo-like manifestation associated with GVHD **(F)**. The slit lamp photograph **(G)** and corneal fluorescein staining photograph **(H)** at the time of first presentation to ophthalmology department in Case 5 were presented. Slit lamp photographs at the time of corneal perforation **(I)** and post-keratoplasty surgery **(J)** in Case 5 were presented. The BCVAs of Case 5 at first presentation, corneal perforation, and last follow-up visit were 20/40, hand motion, and hand motion, respectively.

Representative slit lamp photographs were shown for Cases 4 and 5 who had similar BCVA (20/32 in Case 4 and 20/40 in Case 5) at first presentation and both used topical immunosuppression and corticosteroid eye drops before corneal perforation, whereas Case 4 had the best BCVA (20/40) and Case 5 had the worst BCVA (hand motion) at last follow-up visit among these affected eyes (**Figure 2**). Both patients only had skin GVHD at first visit in ophthalmology, whereas Case 5 had vitiligo-like manifestation associated with GVHD, diffuse corneal staining, and severe blepharitis including hyperemia, pachynsis, and irregularity of palpebral margin. The durations between oGVHD diagnosis and corneal perforation were both short in Cases 4 (2 months) and 5 (3 months). At the time of corneal perforation, BCVA in Case 4 (20/100) was much better than Case 5 (hand motion) resulting from the size and location of corneal lesion. The perforation lesion was located in peripheral cornea and the size of keratoplasty was 4.00 mm in Case 4, whereas the perforation lesion affected the central pupillary zone of cornea and the size of keratoplasty was 6.00 mm in Case 5. Notably, Case 4 underwent CFC surgery at the time of corneal perforation. Five months later, keratoplasty was performed because of conjunctival flap retraction and corneal dissolution progression.

## Discussion

oGVHD is a common complication in HSCT patients, whereas progression to corneal perforation is uncommon and only reported in a few of case reports, where both eyes may be involved (10). The incidence of corneal perforation in oGVHD patients was about 0.7–1.6% in previous studies (4, 8). Considering only seven out of 198 oGVHD patients (3.54%) suffered corneal perforation in our study within more than 9 years, this complication is relatively rare and challenging. The clinical features and effective treatments of this devastating complication are little known and lack of consensus currently, which demonstrates the significance of studying these patients.

oGVHD can be the only target organ of GVHD but often accompanies other GVHD organs. Eyes involvement in GVHD usually indicates a high incidence of morbidity or mortality (11). Patients with multi-organ GVHD including skin and lung disease have increased risk for oGVHD (12). Skin involvement in chronic GVHD patients presents with different non-sclerotic and sclerotic phenotypes. The most typical eruptions in non-sclerotic cutaneous GVHD are a lichen planus-like eruption and poikiloderma, including skin atrophy, pigmentary changes, and telangiectasia (13). All of the seven corneal perforation patients in this study suffered multi-organ GVHD, and six of them had skin involvement. Multi-organ GVHD involvement, especially skin, may be the hallmark of severe oGVHD patients and indicate the poor prognosis for oGVHD.

oGVHD, representing as dry eye disease, is featured by chronic inflammation and pathogenic fibrosis on the ocular surface (6). Fibrosis of lacrimal and meibomian glands, as well as the reduction of conjunctival goblet cells, are the leading causes of dry eye (1, 5, 14). In addition, immune-mediated keratoconjunctivitis gradually aggravate the damage in microenvironment of ocular surface. Blepharitis is likely secondary to the inflammatory ocular surface disease (15). Considering the BUT and Schirmer’s test at first presentation to ophthalmology were terrible in most corneal perforation patients in this study, which were similar to other severe oGVHD patients; there was no remarkable value of BUT or Schirmer’s test for the corneal perforation development assessment of oGVHD patients. All the seven patients presented with worse blepharitis such as eyelid marginal hyperemia or irregularity at the time of corneal perforation than other oGVHD patients without corneal perforation. The vision acuity at first visit in ophthalmology was similar in Cases 4 and 5, whereas Case 5 had diffuse corneal staining, and worse blepharitis including hyperemia, pachynsis, and irregularity of palpebral margin, which may signal worse visual outcomes. Notably, compared with Case 4, the corneal ulceration in Case 5 was more central and larger, resulting in more devastating visual outcome. Consequently, blepharitis may be involved in the inflammation of ocular surface and needed to be carefully assessed during follow-up duration. The patients involved in both cutaneous GVHD and blepharitis indicate the aggressive development of oGVHD.

The duration between HSCT and corneal perforation was usually long with 21 (range 17–145) months as median interval, whereas the duration between oGVHD diagnosis and corneal perforation was relatively shorter with 4 (range: 2–81) months as median interval. Similarly, averages between HSCT and corneal perforation were around 24–26 months in other studies (16). It is unknown whether the aggressive development was due to biological or social factors such as delay in seeking medical care or paying more attention to systemic symptoms and neglecting ocular manifestations. It is suggested to take actions in early diagnosis, long-term follow-up, and multi-disciplinary treatments for oGVHD patients.

Systemic or topical use of corticosteroids and immunosuppressor remains essential for controlling active oGVHD (17). Four patients (57.14%) in this study utilized both immunosuppression and corticosteroid eye drops in the 2 months prior to corneal perforation. Cases 1 and 6 utilized topical immunosuppression and corticosteroid eye drops, respectively. Topically applied corticosteroid can dramatically worsen corneal ulceration through retarding epithelial healing and potentiate collagenases (18). However, the association between topical corticosteroids use and persistent epithelial defect have not been verified (19). Meanwhile, systemic immunosuppression and corticosteroid are important for minimizing the risk of GVHD and reducing the chance of reoperations (20). Consequently, the use of topical corticosteroids should be carefully considered, especially in patients with corneal epithelial defects and stromal thinning.

Besides conservative treatments, surgeries are necessary for corneal perforation patients. Six out of seven corneal perforation patients (85.71%) underwent keratoplasty in this study, except Case 2 with minor peripheral corneal perforation, which only undergoing CFC surgery. Case 4 underwent CFC surgery at the time of corneal perforation. Nevertheless, keratoplasty was performed after 5 months because of conjunctival flap retraction and corneal dissolution progression. Patients who underwent keratoplasty tended to be in more stable condition and have better visual rehabilitation. CFC is also a practical surgery for minor peripheral corneal perforation patients, lack of corneal donors, and patients with surgical contraindication. Clinically manifest tear insufficiency or dry eye disease increases the failure risk of keratoplasty (21). Considering BUT and Schirmer’s test were both terrible in corneal perforation patients, the lack of tear should be intervened in the early stage by autologous serum eye drops, immunosuppression eye drops, and temporary blepharorrhaphy depending on the severity of oGVHD.

The limitations of this study include its retrospective design, limited cases, and incomplete medical record. Further investigations are required to be performed in this rare but vision-threatening complication to investigate the pathogenesis and risk factors of corneal perforation in detail.

## Conclusions

In summary, oGVHD is common in HSCT patients, whereas corneal perforation is uncommon and devastating. Patients involved in both cutaneous GVHD and blepharitis indicate the aggressive development of oGVHD. Early diagnosis, long-term follow-up, and effective multi-disciplinary treatments for oGVHD patients are essential. The use of corticosteroids and immunosuppressor remains essential for controlling oGVHD, whereas the use of topical corticosteroids should be carefully considered in corneal ulceration patients. In addition, appropriate surgeries should be performed to control oGVHD development in time.

## Data availability statement

The original contributions presented in the study are included in the article/**Supplementary Material**. Further inquiries can be directed to the corresponding author.

## Ethics statement

Study procedures and consent to participate of this research were approved by the ethics committee of Dushu Lake Hospital Affiliated to Soochow University (Suzhou, China) (ID: 220053). Written informed consent to participate in this study was provided by the participants’ legal guardian/next of kin. Written informed consent was obtained from the individual(s) for the publication of any potentially identifiable images or data included in this article.

## Author contributions

The work was performed in co-operation with all authors. X-FZ defined research topics and discussed analysis. YX drafted the manuscript, analyzed data and illustrated the results. Y-MW, Z-TS, and X-LY assisted in performing data collection, statistical analysis and reference collection. X-YZ, Y-RR, and Y-JC assisted in data collection and reference collection. FC, XM, and X-WT co-worked on statistical analysis. All authors read and approved the final manuscript.

## Funding

Key Medical Support Disciplines of Suzhou (SZFCXK202123) and Suzhou Medical-Industrial Collaborative Innovation Research Project (SZM2021015).

## Conflict of interest

The authors declare that the research was conducted in the absence of any commercial or financial relationships that could be construed as a potential conflict of interest.

## Publisher’s note

All claims expressed in this article are solely those of the authors and do not necessarily represent those of their affiliated organizations, or those of the publisher, the editors and the reviewers. Any product that may be evaluated in this article, or claim that may be made by its manufacturer, is not guaranteed or endorsed by the publisher.

## Supplementary material

Supplementary Figure 1 Histopathology of corneas after keratoplasty. **(A)** A small amount of squamous epithelium and collagen fibrous stroma were seen in Case 4. **(B)** Hemorrhage and inflammatory cell infiltration were seen in Case 5. **(C)** Collagenization of a small amount of fibrous tissue was seen in Case 7.

## Abbreviations

HSCT, hematopoietic stem cell transplantation; GVHD, graft-versus-host disease; oGVHD, ocular graft-versus-host disease; CFC, conjunctival flap covering; BUT, breakup time of tear film; BCVA, best-corrected visual acuity; ALL, acute lymphoblastic leukemia; NHL, non-Hodgkin lymphoma; AML, acute myeloid leukemia; CML, chronic myeloid leukemia.
