# Supplementary figures and images for: Corneal perforation associated with ocular graft-versus-host disease
Source: Front Oncol. 2022 Sep 15;12:962250. doi: 10.3389/fonc.2022.962250 (PMC9521353; doi:10.3389/fonc.2022.962250)

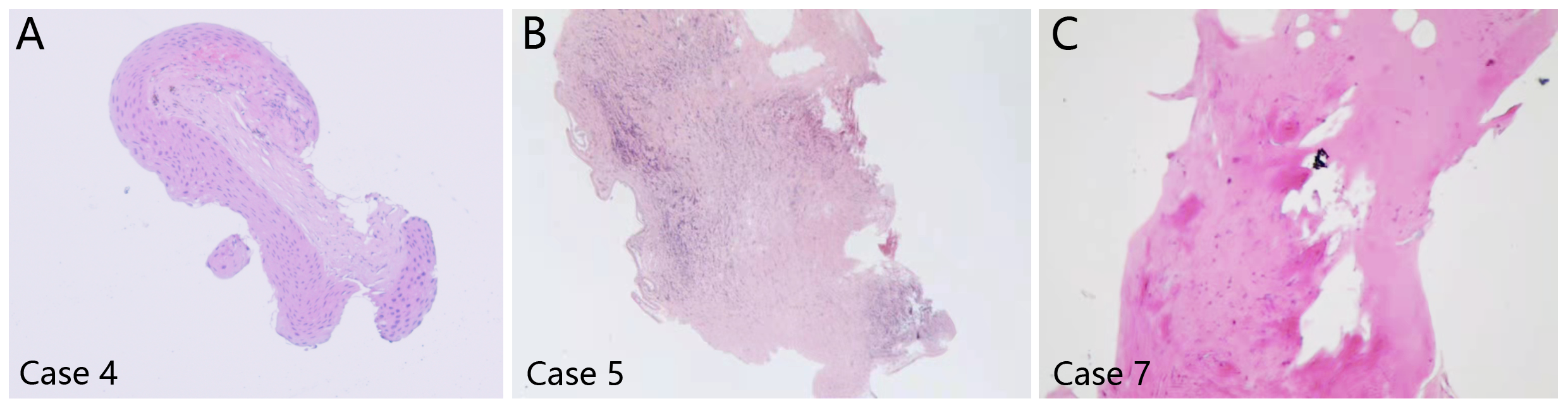

Supplement: Supplementary Figure 1 — Histopathology of corneas after keratoplasty. (A) A small amount of squamous epithelium and collagen fibrous stroma were seen in Case 4. (B) Hemorrhage and inflammatory cell infiltration were seen in Case 5. (C) Collagenization of a small amount of fibrous tissue was seen in Case 7. [file Image_1.tif]
